# Development of a rapid sensor system for nitrate detection in water using enhanced Raman spectroscopy

**DOI:** 10.1039/d4ra08516g

**Published:** 2025-02-20

**Authors:** Xintao Xia, Guiyan Yang, Hongwu Tian, Fengjing Cao, Fan Luo, Daming Dong

**Affiliations:** a School of Mechanical Engineering, Guangxi University Nanning China; b Research Center of Intelligent Equipment, Beijing Academy of Agriculture and Forestry Sciences Beijing 100097 China miketian007@163.com; c Key Laboratory of Agricultural Sensors, Ministry of Agriculture and Rural Affairs Beijing 100097 China

## Abstract

Nitrate is a primary source of nitrogen pollution in aquatic environments, making timely monitoring of its levels in surface and drinking water essential for environmental protection and public health. Conventional laboratory methods are time-consuming and require specialized expertise, while chemical electrode-based online detection systems are hindered by challenges—such as frequent calibration and ion cross-interference—which limits their suitability for long-term monitoring. To address these limitations, a novel nitrate detection method, utilizing an enhanced Raman spectroscopy device, was developed to rapidly detect nitrate in water. The incorporation of an optical feedback mechanism significantly improved detection sensitivity, achieving a detection limit of 2.89 mg per L N, with single sample analysis completed in under one minute. Furthermore, a compact and portable detection system was designed by integrating the Raman enhancement device with a handheld Raman spectrometer, which was successfully validated using real-world environmental water samples. The proposed nitrate detection system features a streamlined user design and user-friendly operation, offering an innovative approach for rapid water pollution detection and early warning. It also provides a foundation for establishing continuous online monitoring systems for water quality.

## Abstract 2

A portable Raman spectrometer combined with enhanced attachment enables sensitive, rapid on-site nitrate detection in water, offering a simple, low-cost solution for environmental water pollution screening.

## Graphical abstract

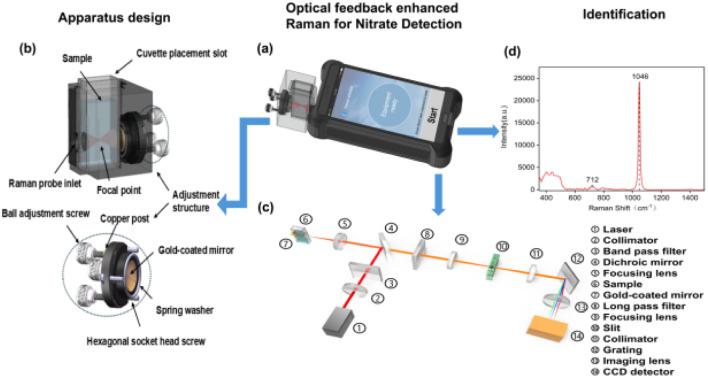

## 1. Introduction

Nitrate, an essential nitrogen compound in ecosystems, plays a vital role in plant physiological and biochemical processes, while also influencing human health through food and drinking water intake.^1,2^ As an essential nutrient, nitrate supports healthy plant growth *via* uptake from naturally transformed soil nitrate and artificial chemical fertilizers. Human consumption of nitrate-rich foods—such as grains, vegetables, and fruits—contributes to physiological benefits, including improved blood flow, reduced blood pressure, and cardiovascular disease prevention.^3–5^

However, excessive nitrate use driven by anthropogenic activities has caused cumulative environmental and health impacts.^6–8^ In agriculture, overapplication of nitrate-based inorganic fertilizers leads to nitrate accumulation in the soil, and subsequent leaching into surface water and groundwater water, through irrigation and rainfall, results in water contamination.^7,9^ Industrial applications of nitrates as raw material for chemical products also contribute to environmental degradation *via* nitrate-laden wastewater discharges.^10^ Excessive nitrate levels can trigger eutrophication, leading to algal blooms and oxygen depletion, which in turn severely impact fish mortality. Moreover, elevated nitrate concentrations in drinking water are linked to health risks such as diabetes, thyroid disease, Parkinson's disease and infantile blue-baby syndrome.^2,11,12^ To mitigate these risks, organizations such as the World Health Organization (WHO) and the United States Environmental Protection Agency (EPA) have set the maximum allowable nitrate concentration in drinking water at 50 mg per L NO_3_^−^. In contrast, China's national drinking water quality standard (GB5749-2022) stipulates a concentration limit of 10 mg per L NO_3_^−^-N.^13–15^ These regulations highlight the critical importance of timely detection and monitoring of nitrate levels in surface water and drinking water to ensure ecological safety and protect human health.

Ultraviolet spectrophotometry, ion chromatography, and chemiluminescence flow injection are commonly employed laboratory techniques for detecting nitrate in water. By using specialized analytical instruments, these methods can achieve good detection accuracy and low detection limits. However, the complexities associated with sample preparation, need for professional operation, and the risk of chemical reagent contamination limit their suitability for rapid field testing. Their lack of timeliness further restricts their effectiveness for routine water quality monitoring and rapid early warning of water pollution.^16–18^

Electrochemical detection methods, such as ion-selective electrodes (ISE) and ion-sensitive field-effect transistors (ISFET), enable online nitrate monitoring.^19,20^ However, these approaches face significant challenges in practical applications, including periodic calibration to address electrode response drift and interference from other ions. As such, developing nitrate detection technologies and equipment that offer rapid on-site detection and stable, reliable performance is of significant practical value and holds promising application potential.^21,22^

Raman spectroscopy, a non-destructive detection technique, operates on the principle of inelastic scattering between the laser light and sample molecules. This interaction provides detailed information about the chemical bonds and functional groups within a sample, enabling both qualitative and quantitative analysis.^23,24^ The technology is broadly applied in fields such as agricultural, food safety, biomedicine, petrochemicals, and environmental testing; and is effective for analyzing solids, liquids, and gases.^25–27^

One of its advantages is that the weak Raman properties of water ensure that Raman spectra are largely unaffected by water molecules, making the technique particularly suitable for the rapid detection of liquid samples. However, detecting target analytes at low concentrations in water remains challenging. Previous studies using conventional Raman spectroscopy with a 532 nm excitation wavelength for quantitative analysis of ions in NaNO_3_ solutions achieved a detection limit of 0.9 mM (approximately 55.81 mg L^−1^).^28^ This sensitivity falls short of the detection standards set by both the EPA and China's regulatory requirements. Thus, optimizing the Raman detection system to enhance the sensitivity of trace nitrate detection in water remains an important area of research requiring further development and innovation.

Conventional Raman spectroscopy only collects unidirectional scattered signals, which limits the full utilization of the scattered light information.^29^ To address this, high-reflectivity optical components, such as gold, silver, and aluminum-coated mirrors have been introduced.^29^ These components facilitate multiple reflections of the incident light within the sample region, enhancing secondary photon focusing and enabling bidirectional Raman signal collection, effectively increasing the intensity of Raman scattering signals.

In biological tissue and cell analysis, replacing traditional CaF_2_ substrates with mirror-grade stainless steel substrates allows the laser beam to undergo secondary interaction with the sample. The reflected laser photons and forward-scattered Raman signals are redirected to the collecting optics, achieving a 1.43-fold increase in average signal intensity for tissue analysis and a 1.64-fold increase for cell analysis.^30^ Similarly, integrating a parabolic mirror into a conventional Raman microscope and adjusting the laser emission angle have successfully enhanced the Raman signals for various samples—hydrotalcite (HT), crystalline silicon, Rhodamine 6G, and bacon fat—by utilizing additional scattering within the sample medium. This approach resulted in signal enhancement ranging from 3.15 to 4.24 times.^31^

Furthermore, a novel method utilizing a curved high-reflection mirror combined with a vertical measurement approach has been developed. This configuration provides broadband optical feedback for both the excitation light and Raman scattered light, enabling efficient reverse reflection of excitation light collection of forward-propagating Raman signals. This method achieved approximately 5.6-fold Raman signal enhancement when detecting pure ethanol and R6G molecules in water.^29^

While these studies represent significant advancements in Raman signal enhancement, practical applications remain constricted by certain limitations. First, the systems are relatively large, leaving room for improvement in portability and flexibility. Additionally, some designs require precise mirror alignment and rely on auxiliary equipment, such as long manipulators, which can pose operational challenges for rapid on-site inspections.

Herein, a miniaturized Raman spectroscopy enhancement device has been designed and developed for the rapid detection of low-concentration nitrates in liquid samples. This device, which is integrated with a handheld Raman spectrometer, forms a compact and efficient system for rapid detection of nitrates in water. Structural optimization of the enhancement device and refinement of detection parameters have significantly improved its sensitivity to nitrates in liquid samples. A quantitative calibration curve for nitrates was established, and the system's performance was validated using both environmental and spiked water samples. The detection limit achieved meets the national standards for nitrate content in drinking water. With its compact design and ease of operation, this system provides an innovative solution for rapid inspection and early warning of nitrate pollution in surface and drinking water, addressing critical needs in environmental protection.

## 2. Materials and methods

### 2.1. Materials

Solid potassium nitrate (KNO_3_) and ethanol (≥99.5%) were obtained from Sigma-Aldrich (Shanghai, China), while Rhodamine 6G (95%) was purchased from Aladdin (Shanghai, China). All reagents were of analytical grade and used directly without further purification. Nitrate solutions were made by dissolving the KNO_3_ in deionized water. A total of 11 nitrate solution samples, with concentrations ranging between 5 and 100 mg L^−1^, were prepared for establishing a quantitative model of nitrate nitrogen (NO_3_^−^-N) and conducting spiked experiments with actual river water samples. In addition, a 0.5 g L^−1^ Rhodamine 6G solution and a 10% ethanol solution were prepared by dissolving the respective analytical-grade reagents in deionized water. These solutions were used to evaluate the experimental device's spectral signal enhancement effect.

Regarding the handling of the samples, we collected them in clean, pre-rinsed containers and immediately filtered them through a filter to prevent any changes in composition. The samples were then stored in a refrigerator at 4 °C to preserve their quality until analysis, which was conducted within 24 hours.

### 2.2. Measuring system structure

**Fig. 1:**
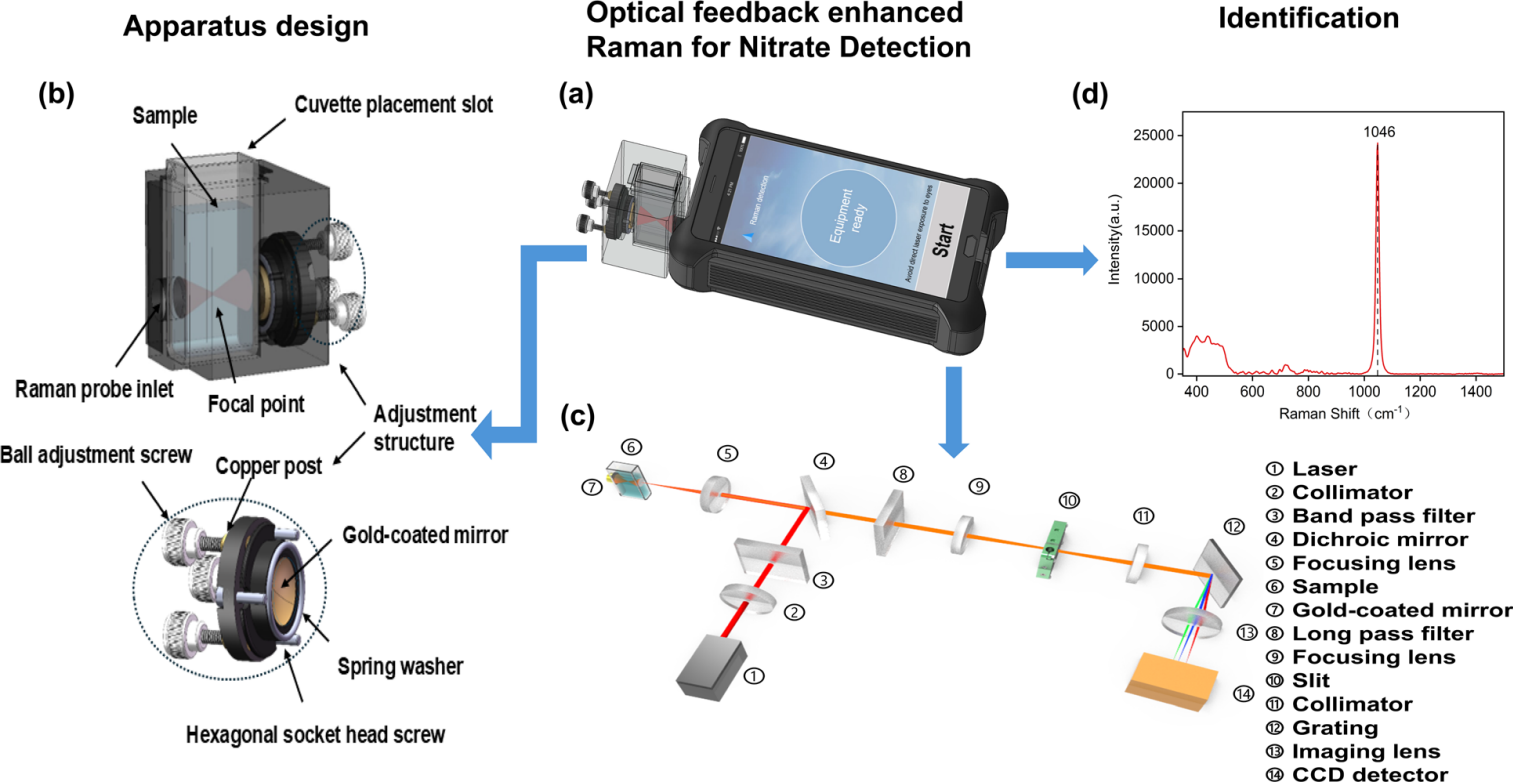
(a) Schematic of the miniaturized liquid sample Raman spectroscopy enhancement detection system. (b) Detailed structure of the miniaturized Raman spectroscopy enhancement device. (c) Optical path structure of the intelligent handheld Raman spectrometer. (d) Raman spectrum of nitrate nitrogen (NO_3_^−^-N) in the wavenumber range of 1000–1400 cm^−1^.

The miniaturized liquid sample Raman spectroscopy enhancement detection system devised in this study (Fig. 1(a)) has compact dimensions of 210 mm × 80 mm × 40 mm. The system is comprised of a handheld Raman spectrometer, a liquid sample spectral enhancement device, an enhancement effect regulator, and a glass cuvette sample cell. The enhancement device itself measures 50 mm × 40 mm × 40 mm, and its detailed structure is shown in Fig. 1(b). Key components include a cuvette cell, a Raman spectroscopy detection interface, a concave reflector, and a reflector adjustment mechanism. The cuvette cell secures the glass cuvette, ensuring the accuracy of the sample positioning. The Raman spectroscopy detection interface aligns with the handheld Raman spectrometer's detector interface, providing stability and accurate signal collection during detection. The enhancement effect regulator consists of ball adjustment screws, copper columns, spring washers, and inner hexagonal cylindrical screws. These components allow for precise adjustments to the reflector's position and angle, optimizing the reflection and enhancement of Raman signals. The glass cuvette, with a path length of 20 mm, holds the liquid sample for analysis.

The intelligent handheld Raman spectrometer measures 162 mm × 80 mm × 35 mm. Its optical path structure is depicted in Fig. 1(c). The handheld Raman spectrometer used in this study is the M1 model that Beijing CloudSpect Technology Co., Ltd manufactured. A 785 nm laser passes through a collimator and a bandpass filter, before being focused onto the nitrate liquid sample *via* a dichroic mirror, generating Raman scattered light. A focusing lens then converts the Raman scattered light into a parallel beam. The dichroic mirror reflects and filters most of the elastically scattered light, while the Raman signal smoothly passes through a long-pass filter. The signal is subsequently focused by a lens onto the spectrometer's slit. After being separated into wavelengths by a grating, the Raman scattered light converges onto the charged coupled device (CCD) for capture and analysis.

The spectrometer's built-in application controls laser emission and CCD data acquisition and processing. Device specifications include: laser wavelength = (785 ± 0.02) nm, focal length = 10 mm, maximum laser power = 500 mW, wavenumber detection range = 200–1800 cm^−1^, and spectral resolution = 8–11 cm^−1^. The spectrometer operates in backscattering mode to collect Raman signals. During operation, the probe connects to the detection setup interface, and a simple click on the “Start” button initiates automatic detection, enabling rapid acquisition of Raman spectral data from the sample.

The liquid sample enhancement device is precisely interfaced with the handheld Raman probe *via* the front circular hole, ensuring a secure connection between the device and the spectrometer. The cuvette is centred within the enhancement device, with its outer wall closely aligned to the Raman probe. The concave reflector is positioned at the rear of the cuvette. During measurement, the laser emitted by the Raman spectrometer is focused onto the liquid sample, generating Raman scattering. The backscattered signal is directed back into the optical path and detected. The excitation light is re-focused into the sample by a gold-plated concave mirror, enabling re-excitation, while the concave mirror also collects forward scattered light. Simultaneously collecting both forward and backward scattering signals enhances signal collection efficiency. The ball adjustment screws and spring washers allow flexible adjustment of the mirror's angle and position, accommodating focus shifts caused by varying liquid refractive indices and optimizing signal enhancement. A gold-plated plano-concave spherical mirror with a 5 mm focal length was selected based on the laser focusing position and cuvette size to ensure precise alignment of the optical axis with the detection optical path—thus maintaining stable signal transmission. The gold-plated mirror exhibits high reflectivity in the near- and mid-infrared bands—including the commonly used 785 nm and 1064 nm laser wavelengths. It provides favourable stability, and oxidation and corrosion resistance, ensuring sustained high performance over time.

### 2.3. Spectral acquisition and data processing

The spectral acquisition parameters were set with a laser power of 500 mW and an integration time of 30 seconds. Three replicate measurements were taken for each sample, and the average value was used as the representative Raman spectrum for subsequent data processing and analysis. These parameter settings were selected to meet the detection requirements for low concentrations of nitrates in water samples. Given the relatively weak Raman signal for nitrates, increasing the laser power and extending the integration time improved the signal-to-noise ratio, thereby enhancing the detection sensitivity.

To accurately determine the detection limit, eight spectral acquisitions of blank water samples were performed. Standard deviations were calculated to assess the method's reliability and repeatability under low-concentration conditions. While taking measurements, dust-free paper was used to carefully clean the device, ensuring precise measurement of the target content and removing any residual liquid before each Raman spectrum acquisition. To maintain consistent measurement conditions, a pipette was employed to control the liquid level in the cuvette, ensuring uniformity across samples.

The Raman spectra of nitrate, Rhodamine 6G, and ethanol are prone to fluorescence interference, making baseline correction essential. This process effectively eliminates the fluorescence background and recovers the true signal. Notably, the high fluorescence background of Rhodamine 6G presents a particular challenge for correction. Additionally, nonlinear baseline drift in the spectra of ethanol and nitrate can obscure important characteristic peaks, making baseline correction equally crucial.

In this study, adaptive iterative reweighted penalized least squares (airPLS) was used for baseline correction on Raman spectra in the 200–1800 cm^−1^ range. This method automatically identifies and removes spectral baselines, ensuring data accuracy.^32^ A univariate linear regression model was developed to estimate NO_3_^−^-N concentration based on the intensity of characteristic Raman peaks and the actual concentrations of nitrate solutions. The model's performance and accuracy were assessed using the Root Mean Square Error (RMSE) and the coefficient of determination (*R*^2^). The detection capability was further evaluated by determining the Limit of Detection (LOD).

## 3. Results and discussion

### 3.1. Materials experimental parameter optimizing

**Fig. 2:**
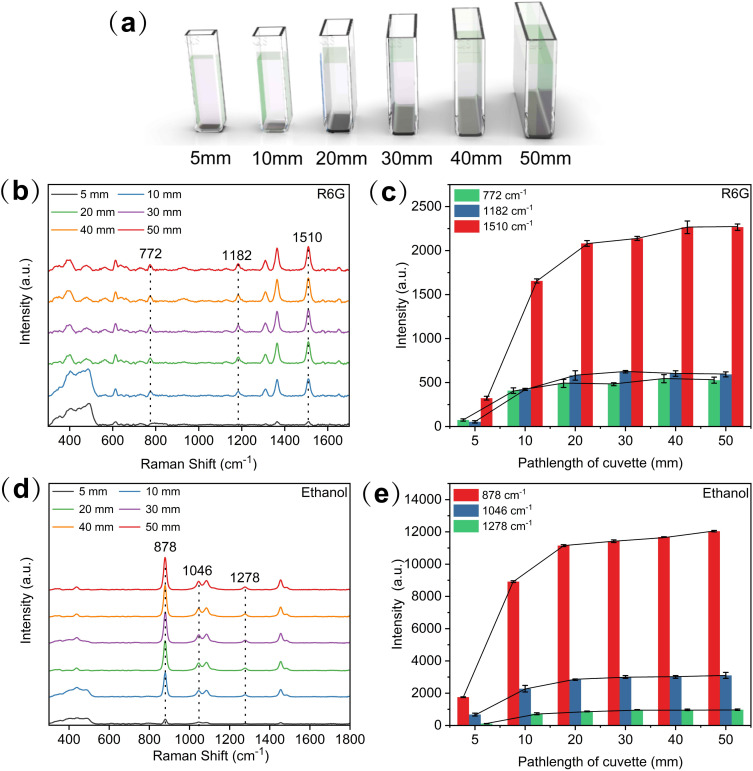
(a) Schematic diagram of cuvettes with different path lengths. (b) Raman spectrum of Rhodamine 6G in cuvettes with different path lengths. (c) Signal intensity variations of Rhodamine 6G under different cuvette path lengths (d) Raman spectrum of ethanol in cuvettes with different path lengths. (e) Signal intensity variations of ethanol under different cuvette path lengths.

The enhanced device designed in this study employed a cuvette to hold the liquid sample. To explore the influence of cuvettes with different path lengths(widths) on Raman signals, cuvettes with path lengths of 5, 10, 20, 30, 40, and 50 mm were tested, as shown in Fig. 2(a). Fig. 2(b) presents the Raman spectra of Rhodamine 6G obtained using these cuvettes, with analyses focusing on the primary peak at 1510 cm^−1^, the secondary peak at 1182 cm^−1^, and the weak peak at 772 cm^−1^. Similarly, Fig. 2(d) shows the Raman spectra of ethanol, highlighting the primary peak at 878 cm^−1^, the secondary peak at 1046 cm^−1^, and the weak peak at 1278 cm^−1^. Fig. 2(c) and (e) illustrate the signal intensity variations in the characteristic peaks for Rhodamine 6G and ethanol under different cuvette path length conditions.

The experimental results show that as the path length of the cuvette increases, the Raman spectral signal's intensity initially rises and then gradually stabilizes. Specifically, the signal intensity obtained with a 5 mm optical path is relatively weak. When the optical path was increased to 10 mm, the signal intensity increased approximately 5-fold. However, further increasing the optical path to 20 mm resulted in only a modest improvement—about 30% higher than that of the 10 mm optical path. For cuvettes with optical path lengths of 30, 40, and 50 mm, the signal intensity remained relatively stable and comparable to that observed with a 20 mm path length. This phenomenon is primarily attributed to the alignment of the laser focus with the sample. Cuvettes with shorter optical paths (*e.g.*, 5 mm) fail to fully align with the laser focus, resulting in incomplete laser energy concentration on the liquid sample and weaker signal generation. Cuvettes with a 10 mm optical path cover more of the laser focus area yet still falls short of fully encompassing it due to the influence of the liquid's refractive index. Longer path lengths (≥20 mm) better align with the laser focus, enabling the Raman spectrometer detector to capture the Raman scattering signals more effectively, thereby improving detection performance. Based on these findings and considering the practical sample volume required for application, a cuvette with a 20 mm path length was selected for subsequent experiments.

**Fig. 3:**
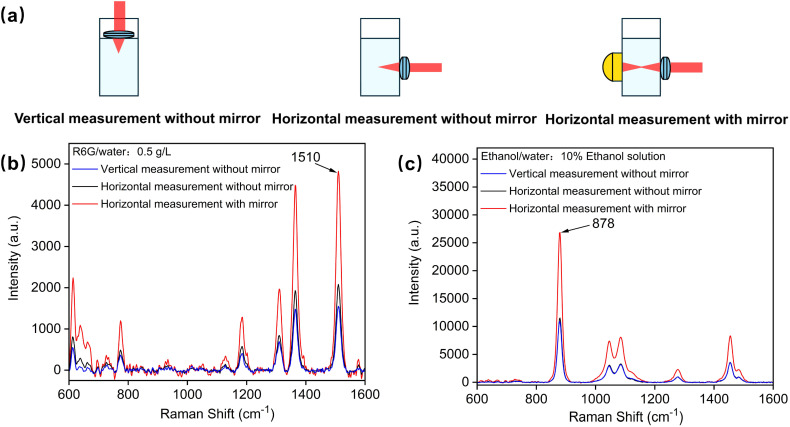
(a) Comparison of measurement configurations using a 20 mm path length cuvette. (b) Signal enhancement of Rhodamine 6G at 1510 cm^−1^ under different measurement configurations. (c) Signal enhancement of ethanol at 878 cm^−1^ under different measurement configurations.

To evaluate the signal enhancement effect of the gold-coated mirror within the liquid sample device, as illustrated in Fig. 3(a), three different measurement configurations were compared using a 20 mm path length cuvette: vertical direct measurement without a mirror, horizontal measurement without a mirror, and horizontal measurement with a rear-positioned mirror. The vertical measurement configuration was included for comparative analysis, because it involves direct laser interaction with the liquid sample, offering a straightforward detection approach with minimal structural requirements for the device.

The samples used in this experiment, Rhodamine 6G and ethanol, were consistent with those analyzed previously. For Rhodamine 6G (Fig. 3(b)), the spectral intensity at the primary peak (1510 cm^−1^) was used to evaluate the signal enhancement factor. Using the enhancement device, the measured intensity at 1510 cm^−1^ was 4826, compared to 2081 for horizontal measurement without the device and 1550 for vertical measurement. This represents a 2.3-fold enhancement compared to horizontal measurement and a 3.1-fold increase compared to vertical measurement. For ethanol, the spectral intensity at the primary peak (878 cm^−1^) sample was analyzed (Fig. 3(c)). The intensity was 26 843 with the enhancement device, compared to 11 341 for horizontal measurement without the device and 10 878 for vertical measurement. This corresponds to a 2.4-fold enhancement over horizontal measurement and 2.5-fold improvement over vertical measurement.

The lower signal intensity observed during vertical measurement can be primarily attributed to the Raman probe's required distance from the liquid surface, limiting its proximity compared to horizontal measurement. In contrast, the horizontal configuration with a rear-positioned mirror facilitates secondary laser focusing and bidirectional signal collection, significantly enhancing the Raman signal intensity. This setup optimizes the signal acquisition path, enabling more effective collection of Raman scattering signals and increasing the spectral intensity.

The experimental results demonstrate that combining horizontal measurement with a concave spherical mirror of appropriate focal length effectively enhances the Raman signal intensity in liquid samples. Furthermore, this method demonstrates good versatility across different sample types.

### 3.2. Quantification model construction

**Fig. 4:**
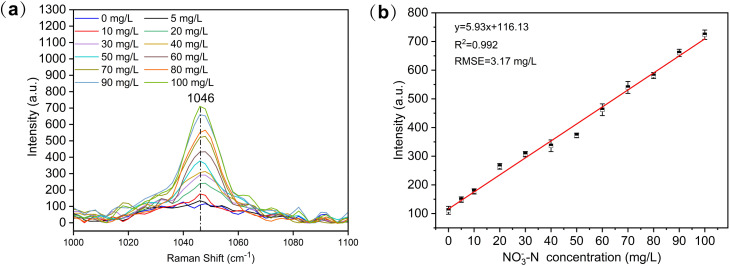
(a) Raman spectrum of nitrate nitrogen (NO_3_^−^-N) standard solution (0–100 mg L^−1^). (b) Calibration curve of NO_3_^−^-N concentration (0–100 mg L^−1^) derived from the Raman peak intensity at 1046 cm^−1^.

Nitrate exhibits two Raman characteristic peaks, located at 1046 cm^−1^ and 718 cm^−1^, respectively, with the 1046 cm^−1^ peak being the primary Raman characteristic peak for nitrate. Therefore, we selected the Raman spectral intensity of NO_3_^−^ at 1046 cm^−1^ to establish the quantitative relationship with concentration.^33^ To validate the designed device's detection capability for NO_3_^−^-N in solution, the Raman characteristic peak intensity at 1046 cm^−1^ was selected for analysis. As shown in Fig. 4, a quantitative relationship between NO_3_^−^-N concentration and signal intensity was established. The calibration curve spans a NO_3_^−^-N concentration range of 0–100 mg L^−1^, with a high *R*^2^ of 0.992 and a RMSE of 3.17 mg L^−1^. The spectral intensity increases with the rise in NO_3_^−^-N concentration. The LOD for NO_3_^−^-N in the standard solution was calculated using eqn (1) and determined to be 2.89 mg L^−1^.^34^1
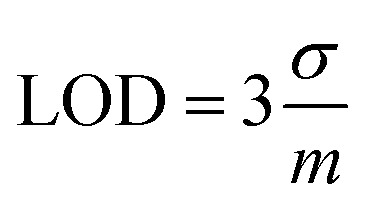
where *σ* denotes the standard deviation of the blank sample, *m* represents the slope of the calibration curve, and “3” corresponds to the confidence interval of 99.86%.

### 3.3. System performance verification

To evaluate the designed system's detection capabilities for real-world applications, river water samples were collected from the Liangshui River (39°48′42′′N, 116°34′02′′E) in Beijing, China, and pond water samples were collected from a pond in Xiaotangshan, Beijing (40°18′05.96′′N, 116°23′29.65′′E). Spiking experiments were performed with NO_3_^−^-N concentrations of 5, 10, 20, and 30 mg L^−1^. Each sample was measured three times, and the average spectra were analysed to assess the model's predictive performance (Table 1).

**Table 1:** The analysis of nitrate nitrogen (NO_3_^−^-N) in spiked samples

Real sample	Sample no.	Original concentration (mg L^−1^)	Added (mg L^−1^)	Founded (mg L^−1^)	Recovery
River water	1	7.02	0	6.89 ± 0.58	106.4%
	2		5	11.23 ± 0.92	101.1%
	3		10	16.64 ± 0.75	93.4%
	4		20	26.29 ± 0.96	93.7%
	5		30	36.24 ± 0.89	95.5%
Pond water	1	3.36	0	3.32 ± 0.19	93.2%
	2		5	7.27 ± 0.67	95.0%
	3		10	15.36 ± 1.05	107.1%
	4		20	22.23 ± 0.86	91.5%
	5		30	31.28 ± 0.57	92.1%

The initial concentration of NO_3_^−^-N in the river water was determined using an ICS-1600 ion chromatograph (Thermo Fisher Scientific), which employs suppressed conductivity detection for precise quantification of anions in aqueous samples. River water and pond water samples were collected using clean polyethylene bottles, filtered through a membrane to remove particulates, and stored at 4 °C in a refrigerator for 24 hours prior to analysis. The measured NO_3_^−^-N concentrations were 7.02 mg L^−1^ for river water and 3.36 mg L^−1^ for pond water. The predicted concentrations using the Raman detection method were 6.89 mg L^−1^ for river water and 3.32 mg L^−1^ for pond water, with relative errors of approximately 1.85% and 1.19%, respectively, compared to the actual values. Recovery rates for NO_3_^−^-N in spiked river water samples ranged from 93.4% to 106.4% and those for pond water samples ranged from 91.5% to 107.1%. These findings demonstrate that the proposed method and device can effectively detect NO_3_^−^-N concentrations in real water samples.

We conducted a comparative analysis of our method against the findings from previous studies, as summarized in Table 2. Our method is simple, portable, and rapid, meeting the standards for surface water pollution detection. SERS is a commonly used signal enhancement method in Raman spectroscopy and has been applied to the detection of many trace and ultra-trace targets, with its outstanding sensitivity being a significant advantage. However, the complex preparation of SERS substrates and poor measurement reproducibility limit its application in practical scenarios.^35^ The detection device proposed in this study is highly integrated, compact, portable, and easy to operate. The physical enhancement method employed ensures the consistency of the device. While this physical enhancement approach provides limited improvement in detection capability from the perspective of the detection limit, it meets the requirements for detecting nitrate pollution in surface water. This makes it a convenient tool for rapid screening of water pollution, offering higher practical value.

**Table 2:** Comparison of our results with previous work

Detection method	NO_3_^−^-N LOD (mg L^−1^)	Linear range (mg L^−1^)	Detection time	Instrument portability	Ref.
Ultraviolet spectrophotometry	Not reported	0.2–2.8	>5 min	Suitable for laboratory use	36
Ion chromatography	0.04	0.1–75	>10 min	Moderately portability	37
Chemiluminescence flow injection	9.8 × 10^−6^	2.8 × 10^−5^–0.14	15 samples per hour	Moderately portable	38
Ion-selective electrodes (ISE)	0.7	0.16–1444.7	>5 min	Suitable for field and lab use	39
Ion-sensitive field-effect transistors (ISFET)	1.2	3–20	60 s requires 3 hours of soaking	Field-deployable with some preparation	40
Raman spectroscopy (portable spectrometer, direct measurement)	12.61	0–1400	30 s	Field-friendly	41
Surface-enhanced Raman spectroscopy (SERS)	Not reported	1–1000	60 s	Portable with specific requirements	42
This work	2.89	0–100	30 s	Highly portable and user-friendly	—

## 4. Conclusions

In this study, a small-scale Raman spectroscopy-enhanced detection device for liquid samples was developed and integrated with a handheld Raman spectrometer to create a rapid on-site detection system for low-concentration nitrates in water. The enhancement device increased the Raman characteristic spectral intensity of NO_3_^−^-N by 2.3 times compared to direct liquid detection. The calibration curve achieved an *R*^2^ and RMSE of 0.992 and 3.17 mg L^−1^, respectively, with a detection limit for NO_3_^−^-N of 2.89 mg L^−1^, fully meeting the national standard for NO_3_^−^-N in drinking water.

The device features a compact structure and user-friendly operation, enabling convenient on-site, rapid detection and screening of nitrate pollution in surface water and drinking water. A single sample can be completed within one minute. Furthermore, with structural improvements, the device has the potential for continuous online monitoring of water samples. The detection device developed in this study offers a novel technical solution for rapid water pollution screening and inspection, thereby effectively addressing future challenges in water quality monitoring.

## Data availability

Data can be available upon request from the authors.

## Author contributions

Xintao Xia: was responsible for the design of the device, the implementation of the experiment, and the visualization of the experimental data. Hongwu Tian: conceived the experimental ideas, proposed the experimental methods, wrote the draft, and modified the final manuscript. Guiyan Yang: contributed to the analysis of experimental data, experimental implementation and supervision. Fengjing Cao: provided guidance and oversight for the design and execution of spiking experiments. Fan Luo: supervision. Daming Dong: conceptualization, methodology.

## Conflicts of interest

The authors declare that they have no known competing financial interest or personal relationships that could have appeared to influence the work reported in this paper.
